# Growth-Arrest-Specific Protein 2 Inhibits Cell Division in *Xenopus* Embryos

**DOI:** 10.1371/journal.pone.0024698

**Published:** 2011-09-09

**Authors:** Tong Zhang, Bama Dayanandan, Isabelle Rouiller, Elizabeth J. Lawrence, Craig A. Mandato

**Affiliations:** 1 Department of Biology, McGill University, Montreal, Quebec, Canada; 2 Department of Anatomy and Cell Biology, McGill University, Montreal, Quebec, Canada; UT Southwestern Medical Center, United States of America

## Abstract

**Background:**

Growth-arrest-specific 2 gene was originally identified in murine fibroblasts under growth arrest conditions. Furthermore, serum stimulation of quiescent, non-dividing cells leads to the down-regulation of *gas2* and results in re-entry into the cell cycle. Cytoskeleton rearrangements are critical for cell cycle progression and cell division and the Gas2 protein has been shown to co-localize with actin and microtubules in interphase mammalian cells. Despite these findings, direct evidence supporting a role for Gas2 in the mechanism of cell division has not been reported.

**Methodology and Principal Findings:**

To determine whether the Gas2 protein plays a role in cell division, we over-expressed the full-length Gas2 protein and Gas2 truncations containing either the actin-binding CH domain or the tubulin-binding Gas2 domain in *Xenopus laevis* embryos. We found that both the full-length Gas2 protein and the Gas2 domain, but not the CH domain, inhibited cell division and resulted in multinucleated cells. The observation that Gas2 domain alone can arrest cell division suggests that Gas2 function is mediated by microtubule binding. Gas2 co-localized with microtubules at the cell cortex of Gas2-injected *Xenopus* embryos using cryo-confocal microscopy and co-sedimented with microtubules in cytoskeleton co-sedimentation assays. To investigate the mechanism of Gas2-induced cell division arrest, we showed, using a wound-induced contractile array assay, that Gas2 stabilized microtubules. Finally, electron microscopy studies demonstrated that Gas2 bundled microtubules into higher-order structures.

**Conclusion and Significance:**

Our experiments show that Gas2 inhibits cell division in *Xenopus* embryos. We propose that Gas2 function is mediated by binding and bundling microtubules, leading to cell division arrest.

## Introduction

Cytoskeletal dynamics are essential for many fundamental cellular processes, including cell division, wound healing and cell motility [1], [2], [3]. During cell division, for example, dramatic rearrangements of the actin and microtubule cytoskeletons are required in order for the cell to change morphology, segregate its chromosomes and execute cytokinesis. The ability of the cytoskeleton to adapt to constant physiological changes is mediated, in part, by actin and microtubule-binding proteins and cross-linking proteins that regulate cytoskeleton dynamics. Many actin-microtubule cross-linking proteins have been identified; however, their functions and mechanisms of regulation remain unclear [4]. One such potential cytoskeleton-interacting protein is the growth-arrest-specific (Gas) 2 protein.

The Gas2 protein belongs to the growth-arrest-specific protein family and is widely expressed in human tissues [5]. Although Gas2 has a putative N-terminal actin-binding calponin homology (CH) domain [6] and a C-terminal tubulin-binding Gas2 domain, no direct evidence for Gas2-cytoskeleton interactions has been reported. However, immunofluorescence studies demonstrated that the full-length Gas2 co-localizes with filamentous actin (F-actin) at the cell cortex and in stress fibers in growth-arrested NIH 3T3 fibroblasts [6] and the Gas2 domain co-localize with microtubules in COS-7 cells [7].

Although the majority of cells in an organism are quiescent, they are able to re-enter the cell cycle and proliferate after stimulation [8]. Several lines of evidences support a role for Gas2 in cell cycle progression. First, the *gas2* gene was originally identified in a genetic screen of murine fibroblasts that were cultured under growth arrest conditions [9]. Second, *gas2* is down-regulated upon serum and growth factor stimulation [6]. Furthermore, the Gas2 protein is phosphorylated on a serine residue at the G_0_ to G_1_ transition allowing quiescent G_0_ cells to re-enter the cell cycle [6]. However, whether Gas2 plays a direct role in the mechanism of cell division and whether this function is mediated by its cytoskeletal binding properties are completely unknown.

In this study, *Xenopus* embryos and oocytes were used to study Gas2 functions in cell division. *Xenopus* embryo undergoes a time-regulated synchronized cell division in the early stages of its development and therefore is a useful *in vivo* model system for studying cell division. An established *Xenopus* oocyte wound-induced contractile array assay, which mimics cytokinesis, was used to study Gas2 interactions with the cytoskeleton *in vivo* [10]. Furthermore, cytoskeleton co-sedimentation assays and electron microscopy were performed to study Gas2-cytoskeletal interactions *in vitro*. Our results suggest that the Gas2 protein plays a role in cell division and that its function is mediated by bundling microtubules.

## Results

### The Gas2 protein is conserved during evolution

**Figure 1:**
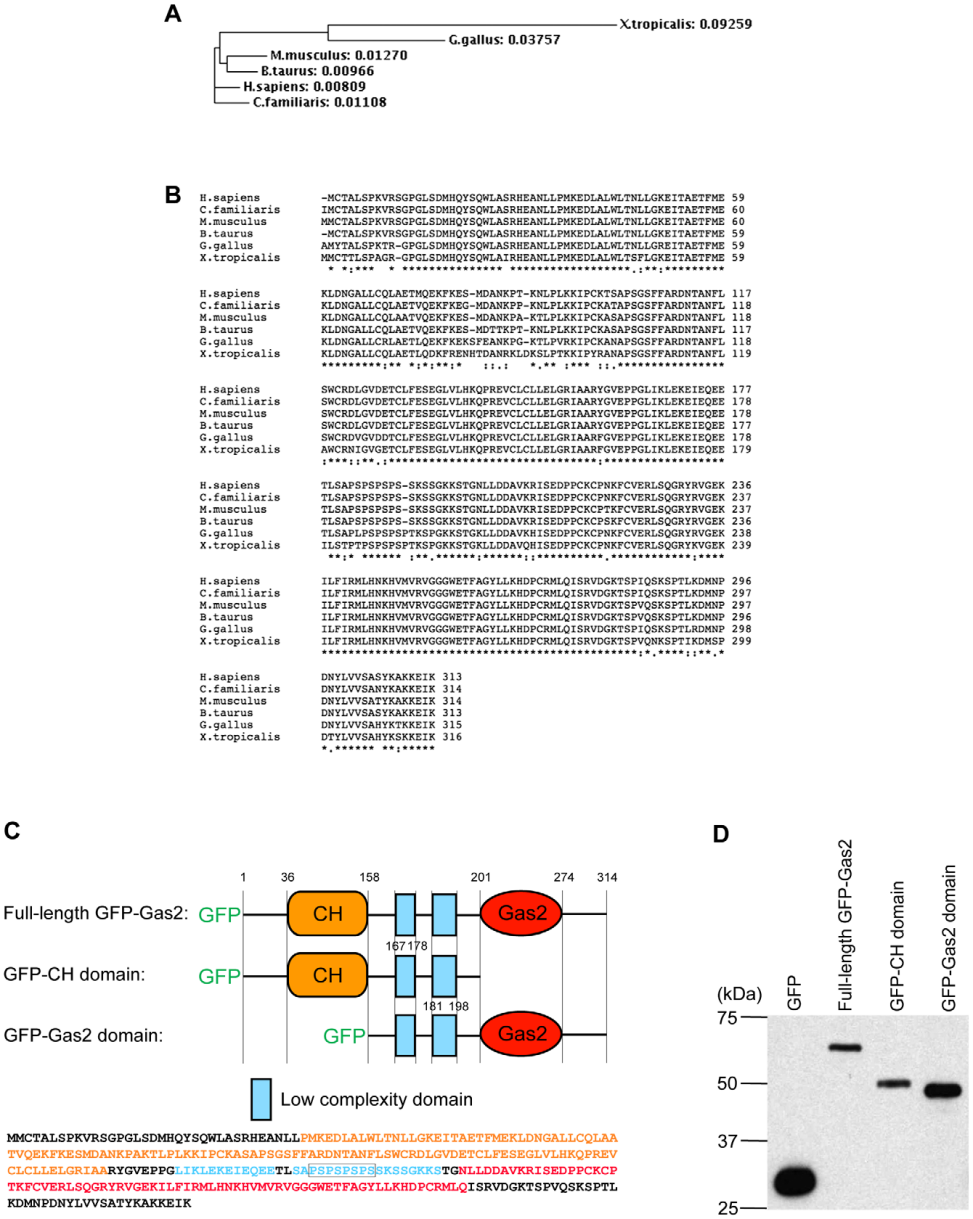
The Gas2 protein is conserved during evolution. (A) Phylogenetic tree of Gas2 protein. The phylogenetic relationship was derived by ClustalW program. The numbers represent the evolutionary distances. (B) Multiple sequences alignment of Gas2 amino acid sequences from different species. The alignment was generated using ClustalW. “*” indicates identical amino acids in all sequences in the alignment; “:” indicates that conserved substitutions have been observed; and “.” indicates that semi-conserved substitutions have been observed. (C) The mouse Gas2 protein [P11862] domain structure and amino acid sequence. N-terminal full-length GFP-Gas2, GFP-CH domain and GFP-Gas2 domain constructs were used in this study. The domains colors are matched with relative amino acid colored sequences. The boxed region indicates the 4 P-S repeats location, which gives this region more structural flexibility. (D) Western blot analysis of GFP Gas2 constructs expression. GFP = 27 kDa, full-length GFP-Gas2 = 62 kDa, GFP-CH domain = 49 kDa and GFP-Gas2 domain = 44 kDa.

Bioinformatics comparisons of the Gas2 protein sequences among *Homo sapiens* (human) [O43903], *Canis familiaris* (Dog) [F1PBV2], *Mus musculus* (house mouse) [P11862], *Bos taurus* (Bovine) [A8E4Q5], *Gallus gallus* (Chicken) [F1NSM4] and *Xenopus tropicalis* (Silurana tropicalis) [ENSXETP00000005555] reveal that Gas2 is conserved during evolution (Fig. 1A and B), which suggests that Gas2 has a conserved biological function. The Gas2 protein contains two cytoskeletal binding domains: a putative actin-binding calponin homology (CH) domain near its N-terminus (Fig. 1C for mouse Gas2 [P11862] amino acids#: 36-158), and a tubulin-binding Gas2 domain near its C-terminus (amino acids#: 201–274). There are two low complexity domains (amino acids#: 167–178 and 181–198) between the CH and Gas2 domains, and the second low complexity domain contains 4 proline-serine (P-S) repeats (amino acids#: 183–190 shown in the boxed region in its sequence), which gives this region more structural flexibility. The N-terminus GFP-tagged full-length mouse Gas2 protein [P11862] and its CH and Gas2 domains were cloned to study their functions in *Xenopus laevis* (Fig. 1C), and the protein expression in *Xenopus* oocytes was verified by Western blot analysis (Fig. 1D).

### Exogenous expression of the full-length Gas2 protein in *Xenopus* embryos inhibits cell division

The Gas2 protein is up-regulated upon serum starvation in NIH 3T3 cells and it also results in cells arresting at G_0_ phase of cell cycle [9]. Conversely, the Gas2 protein level is down-regulated with serum and growth factor stimulation, allowing for cell cycle progression [6]. These two observations imply a relationship between cell cycle progression and the Gas2 protein level. To test this hypothesis, *Xenopus laevis* embryos were used to investigate whether the over-expression of the Gas2 protein could inhibit cell division. The first cell division of *Xenopus* embryo occurs approximately 90 minutes post-fertilization at room temperature, and subsequent cell divisions occur at 30 minute intervals. *Xenopus* embryos are relatively large, approximately 1 mm in diameter, facilitating the microinjection of the Gas2 protein and the observation of cell morphological changes.

**Figure 2:**
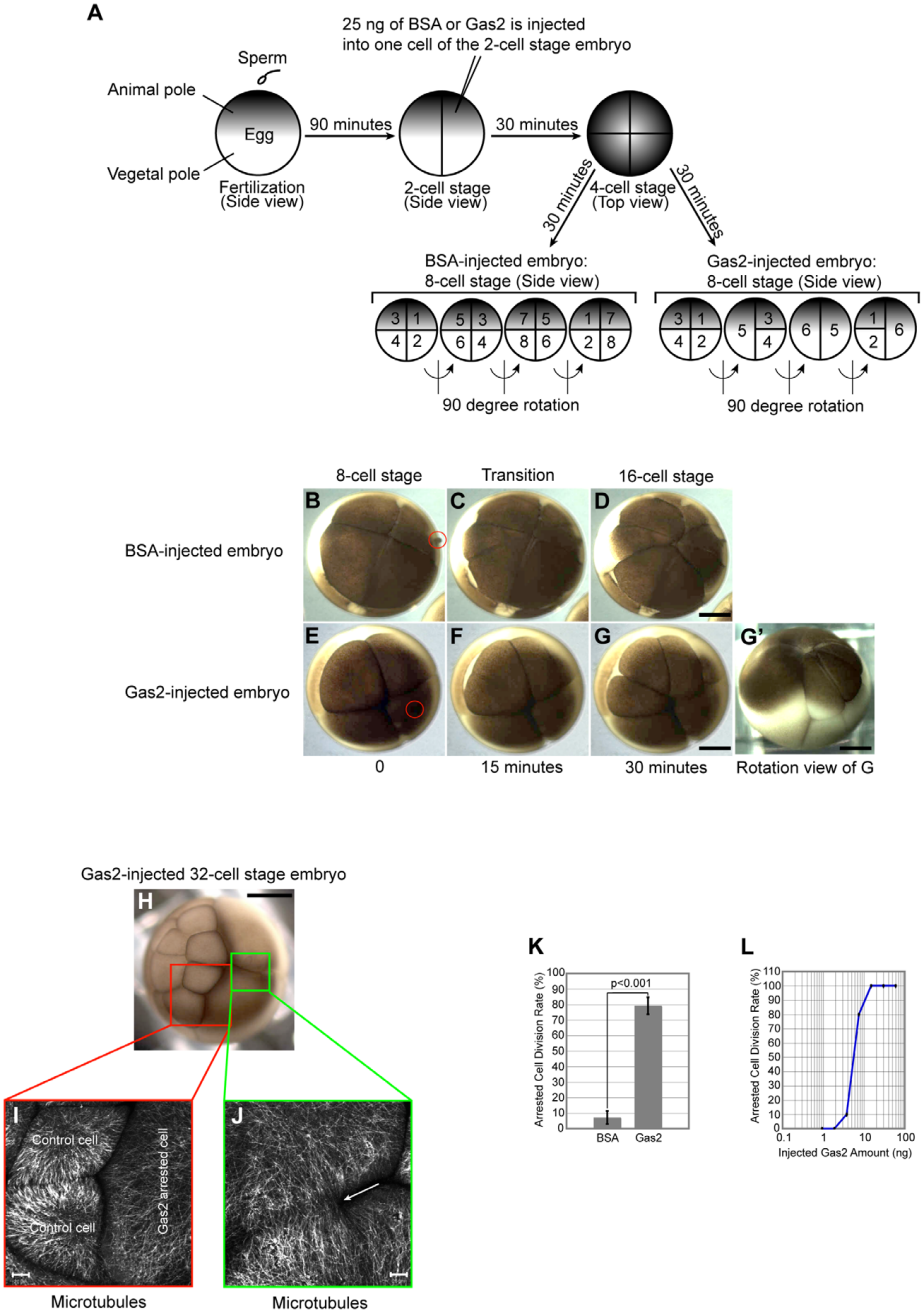
Exogenous Gas2 inhibits cell division in *Xenopus* embryos. (A) The experimental flowchart and schematically represented results. The darker side of the egg represents the animal pole and the lighter bottom side represents the vegetal pole. The 90 degree angle rotation views show cell numbers in BSA-injected control vs. Gas2-injected embryos. (B–D) BSA-injected *Xenopus* embryos continue through normal cell divisions (Video S1). (E–G) Gas2-injected embryo cells divide once, then arrest in subsequent cell divisions. The non-injected control cell of the Gas2-injected embryo divides normally, and the cell number increases 2-fold every 30 minutes at room temperature (Video S2). The red circles in B and E indicate the needle injection sites. Time was set to 0 at 8-cell stage for demonstration purposes. (G') The rotation view of Fig. 2G embryo shows one of the large arrested cells on the left. Bars, 0.3 mm. (H) Stereo microscopy examination of a Gas2-injected embryo at the 32-cell stage. The normal dividing cells are on the left and the two large arrested cells are on the right. Bar, 0.3 mm. (I and J) Confocal microscopy analysis of post-injected embryo cells with tubulin antibody staining. The white arrow in J indicates that the two large, arrested cells remain connected with microtubules. Bars, 20 µm. (K) Statistical analysis of cell division rate in BSA- and Gas2-injected embryos. Only 7.2±4.2% of BSA-injected embryos arrest in cell division; however, 79.2±5.5% of Gas2-injected embryos arrest in cell division (n = 300 embryos and from 5 experiments, p<0.001). (L) Dosage dependent analysis of cell division rate in Gas2-injected embryos. The calculated LD_50_ from the dosage dependent analysis graph is 5.5 ng or 31 µM for one cell of the 2-cell stage *Xenopus* embryo. The x-axis of the graph is in logarithmic scale.

The fertilized eggs were injected after they completed their first cell division. One cell of the 2-cell stage embryo was microinjected with either 25 ng bovine serum albumin (BSA) in phosphate buffered saline (PBS) solution or the bacterial purified full-length mouse Gas2 protein into the cytoplasm of a cell. The non-injected cell of the 2-cell embryo acts as an internal homo-genomic negative control (Fig. 2A). The BSA-injected embryos are shown in Fig. 2B–D (Video S1) and Gas2-injected embryos are shown in Fig. 2E–G′ (Video S2). BSA-injected embryos proceed through normal cell division and the number of cells increased 2-fold every 30 minutes. However, cells that were injected with 25 ng Gas2 protein divided once and then arrested in subsequent cell divisions while the non-injected control cells of the same embryo divided normally (Fig. 2H smaller cells on the left). In Gas2-injected cells, cell division arrested after approximately 30 minutes, consistent with the time required for Gas2 to diffuse in the cytoplasm of the injected cell (approximately 3.5 µm/second under the injection needle pressure). Statistical analysis of the microinjection experiments showed that 7.2±4.2% BSA-injected embryos arrested in cell division, likely as a result of unsuccessful fertilization and/or microinjection damage to the embryos. In contrast, 79.2±5.5% Gas2-injected embryos arrested in cell division, which was a significantly higher percentage than the BSA control group (Fig. 2K). The non-100% arresting effect by Gas2 in cell division is thought to be caused by the Gas2 protein clotting in the injection needle. BSA-injected embryos succeed in developing into tadpoles, but Gas2-injected embryos all died within 24 hours, presumably due to the interference of the arrested cells with the embryo development. In 32-cell embryos, the cortical microtubules were significantly longer in the Gas2-injected arrested cells than non-injected control cells (Fig. 2I). The two large Gas2 arrested cells were connected together with microtubules (arrow in J). Two-cell stage *Xenopus* embryos were microinjected with increasing amounts of the Gas2 protein to investigate the minimum amount of Gas2 required to arrest their cell division. Dosage dependent analysis was done by microinjecting the same volume (5 nl) of a serial dilution of Gas2 protein, and observing the cell division arresting phenotype. The calculated lethal dose 50% (LD_50_) was 5.5 ng or 31 µM for one cell of the 2-cell stage *Xenopus* embryo (Fig. 2L).

### Gas2 co-localizes with microtubules in arrested cells and over-expression of either the full-length Gas2 protein or the Gas2 domain alone results in multinucleated cells

**Figure 3:**
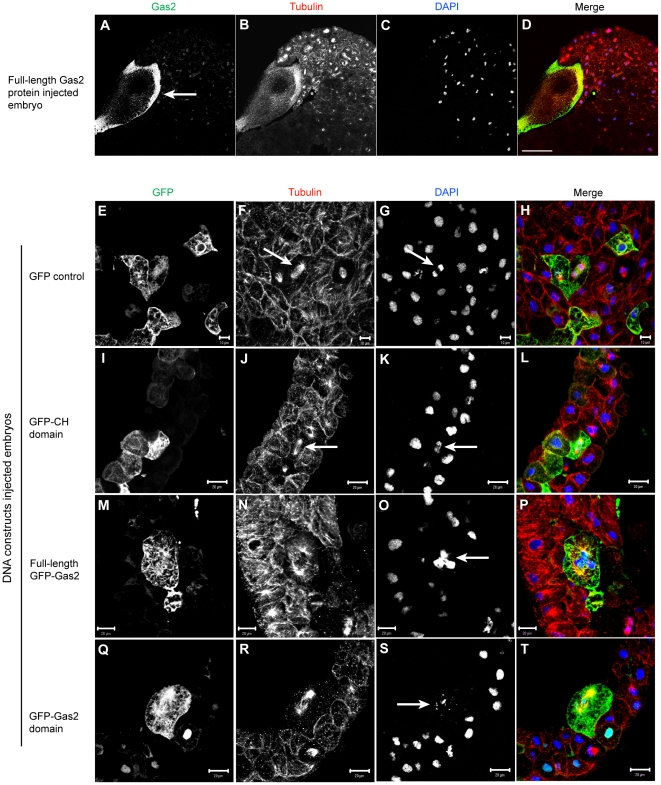
Over-expression of either the full-length Gas2 protein or the Gas2 domain alone arrests cell division in *Xenopus* embryos. (A–D) The Gas2 protein injected cell is relatively larger than non-injected normal dividing cells. Gas2 localizes to the injected cell cortex (arrow in A) and also co-localizes with microtubule network shown in yellow in the merged image D. Bar, 100 µm. (E–H) Cells expressing GFP alone and (I–L) GFP-CH domain are similar in size to the neighboring non-expressing control cells. Cells in anaphase can be recognized by their microtubule morphology (arrows in F and J) and separating chromosomes (arrows in G and K). Bars in E–H, 10 µm and bars in I–L, 20 µm. (M–P) Cells expressing full-length GFP-Gas2 and (Q–T) GFP-Gas2 domain expressing cells are relatively larger than neighboring non-expressing cells and they also have multiple nuclei, indicating a failure in cell division (arrows in O and S). Bars, 20 µm.

To further investigate the mechanism of how Gas2 arrested *Xenopus* embryo cell division, cryo-confocal microscopy was performed with Gas2-injected embryos that were fixed and stained for Gas2, tubulin and DNA by DAPI (Fig. 3A–D). Gas2-injected cells that failed division were larger than non-injected control dividing cell. Gas2 localizes to the cortex in arrested cells and co-localizes with cortical microtubules (arrow in A). The Gas2-injected cell has no DAPI staining presumably due to the fact that the injected cell arrested in the early embryo developmental stage when the cell is relatively large; therefore, it is difficult to maintain the integrity of all cellular organelles (such as the cell nucleus) during cryo-cutting (Fig. 3C shows no DAPI staining in the large arrested cell). The same observations were obtained with repeated experiments.

To examine the exogenous Gas2 during cell division, GFP control, full-length GFP-Gas2, GFP-CH domain and GFP-Gas2 domain constructs were injected into the cytoplasm of one cell of the 2-cell stage embryos, and embryos were fixed at either Stage 10 or 11. Cryo-confocal microscopy was performed to examine post-injected embryos. Embryos expressing the GFP control (Fig. 3E–H) and GFP-CH domain (Fig. 3I–L) are approximately the same size as neighboring non-expressing control cells, which is an indication of normal cell division. Some GFP and GFP-CH domain expressing cells can be recognized in anaphase of mitosis on the basis of their microtubule morphology (arrows in F and J) and their separating chromosomes (arrows in G and K). However, cells expressing both full-length GFP-Gas2 (Fig. 3M–P) and GFP-Gas2 domain (Fig. 3Q–T) are larger than control cells, and have multiple nuclei, indicative of a failure in cell division (arrows in O and S). The full-length GFP-Gas2 (Fig. 3M) and GFP-Gas2 domain (Fig. 3Q) localize to the cell cortex and co-localize with microtubule spindles.

### Gas2 stabilizes microtubules via its Gas2 domain in *Xenopus* oocytes

**Figure 4:**
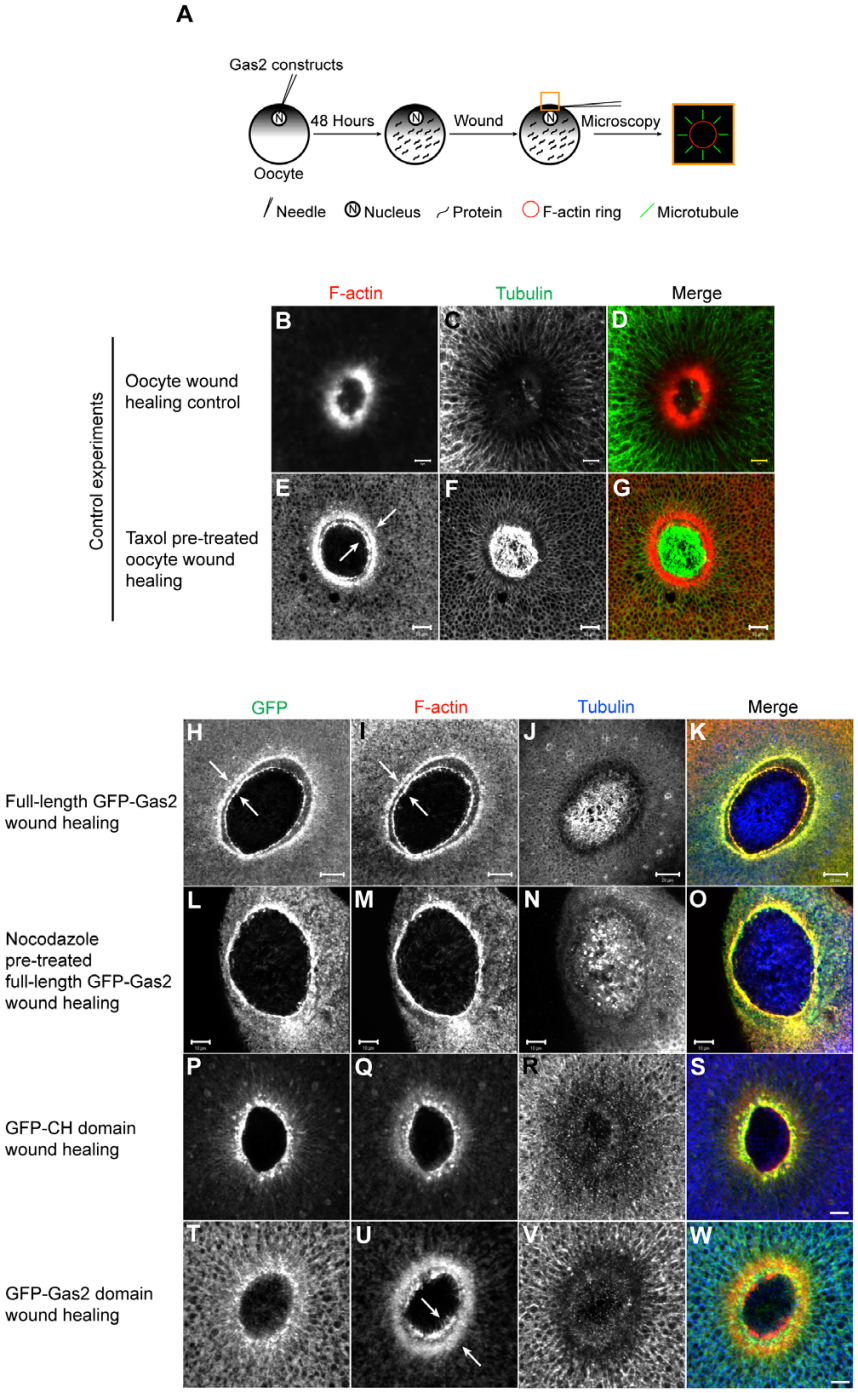
The expression of either full-length Gas2 protein or the Gas2 domain alone results in abnormal double actin rings at the wound border. (A) The experimental flowchart and schematically represented results. Oocytes nuclei were injected with different GFP Gas2 constructs and incubated for 48 hours to allow for protein expression. The animal pole of an oocyte was then wounded, fixed and stained for F-actin and tubulin. The wound site was excised and examined by confocal microscopy. (B–D) Oocyte wound healing control experiment. F-actin forms a single ring and microtubules radially distribute around the wound. Bars, 5 µm. (E–G) Oocytes pre-treated with Taxol form abnormal double actin rings (arrows in E) during wound healing. Bars, 10 µm. (H–K) Oocytes expressing the full-length GFP-Gas2 form double Gas2 rings (arrows in H), which co-localize with double actin rings (arrows in I) during wound healing. Bars, 20 µm. (L–O) Oocytes expressing the full-length GFP-Gas2 and pre-treated with nocodazole form a single Gas2 ring, which co-localizes with single actin ring during wound healing. Bars, 10 µm. (P-S) Oocytes expressing GFP-CH domain alone form a single actin ring during wound healing. Bar, 10 µm. (T-W) Oocytes expressing GFP-Gas2 domain alone form a single Gas2 domain ring, which localizes between the double actin rings (arrows in U) during wound healing. The GFP-Gas2 domain does not co-localize with either actin ring. Bar, 10 µm.

To test the hypothesis that Gas2 regulated microtubule dynamics/stability leading to cell division arrest, *Xenopus laevis* oocyte wound healing contractile array assay, which mimics cytokinesis, was used to study Gas2 interactions with the cytoskeleton *in vivo* [10]. The relatively large size of *Xenopus* Stage VI oocytes (approximately 1 mm in diameter) provides a useful tool for studying cytoskeleton dynamics under the cell surface [11]. The nuclei of Stage VI oocytes were injected with either full-length GFP-Gas2, GFP-CH domain or GFP-Gas2 domain constructs, and incubated for 48 hours to allow for protein expression. A micro-capillary glass tube pulled needle was used to wound the animal poles of the oocytes. After wounding, the oocytes were allowed to recover for approximately 5 minutes in oocyte ringer-2 (OR-2) solution, and were then fixed and stained for F-actin and tubulin (Fig. 4A). The non-injected control oocyte forms a single actin contractile ring around the wound; and microtubules radially distribute around the wound (Fig. 4B–D). In oocytes pre-treated for one hour with 10 µM taxol to stabilize microtubules, actin assembles into two separate rings surrounding the wound (Fig. 4E–G). The internal ring is composed of the contractile F-actin, while the outer ring is formed by *de novo* actin polymerization (arrows in E) [2]. Please see the discussion for further details.

Oocytes expressing full-length GFP-Gas2 have a similar phenotype to taxol-treated oocytes. The GFP-Gas2 rings (arrows in 4H) co-localize with the two separate actin rings (arrows in 4I) during wound healing (Fig. 4H–K). This observation implies that Gas2 mimics taxol-treatment and therefore may stabilize microtubules. When full-length GFP-Gas2-expressing oocytes were pre-treated with 20 µM nocodazole to destabilize microtubules for one hour prior to wounding, GFP-Gas2 co-localized with a single actin ring instead of double rings (Fig. 4L–O); therefore, microtubule stabilization by Gas2 was sensitive to nocodazole treatment. Gas2 has an actin-binding CH domain near its N-terminus, which may explain the observed co-localization of GFP-Gas2 and actin rings. Oocytes expressing GFP-CH domain have a single actin ring during wound healing (Fig. 4P–S). This result is also observed in the control (Fig. 4B–D) and nocodazole-treated oocytes expressing full-length GFP-Gas2 (Fig. 4L–O). GFP-CH domain co-localizes with the actin ring since it is the actin-binding domain of Gas2. Oocytes expressing the GFP-Gas2 domain alone form double actin rings (arrows in 4U), and these double actin rings are similar to the taxol pre-treated (Fig. 4E) and full-length GFP-Gas2 expressing oocytes (Fig. 4I). GFP-Gas2 domain forms a ring-like structure and radially distributes around the wound (Fig. 4T), but it does not overlap with either actin ring. This series of experiments showed that the tubulin-binding Gas2 domain alone is sufficient for eliciting the double actin rings phenotype, suggesting that Gas2 binds microtubules via its Gas2 domain and stabilizes them during oocyte wound healing.

### The Gas2 protein co-sediments with F-actin and microtubules

**Figure 5:**
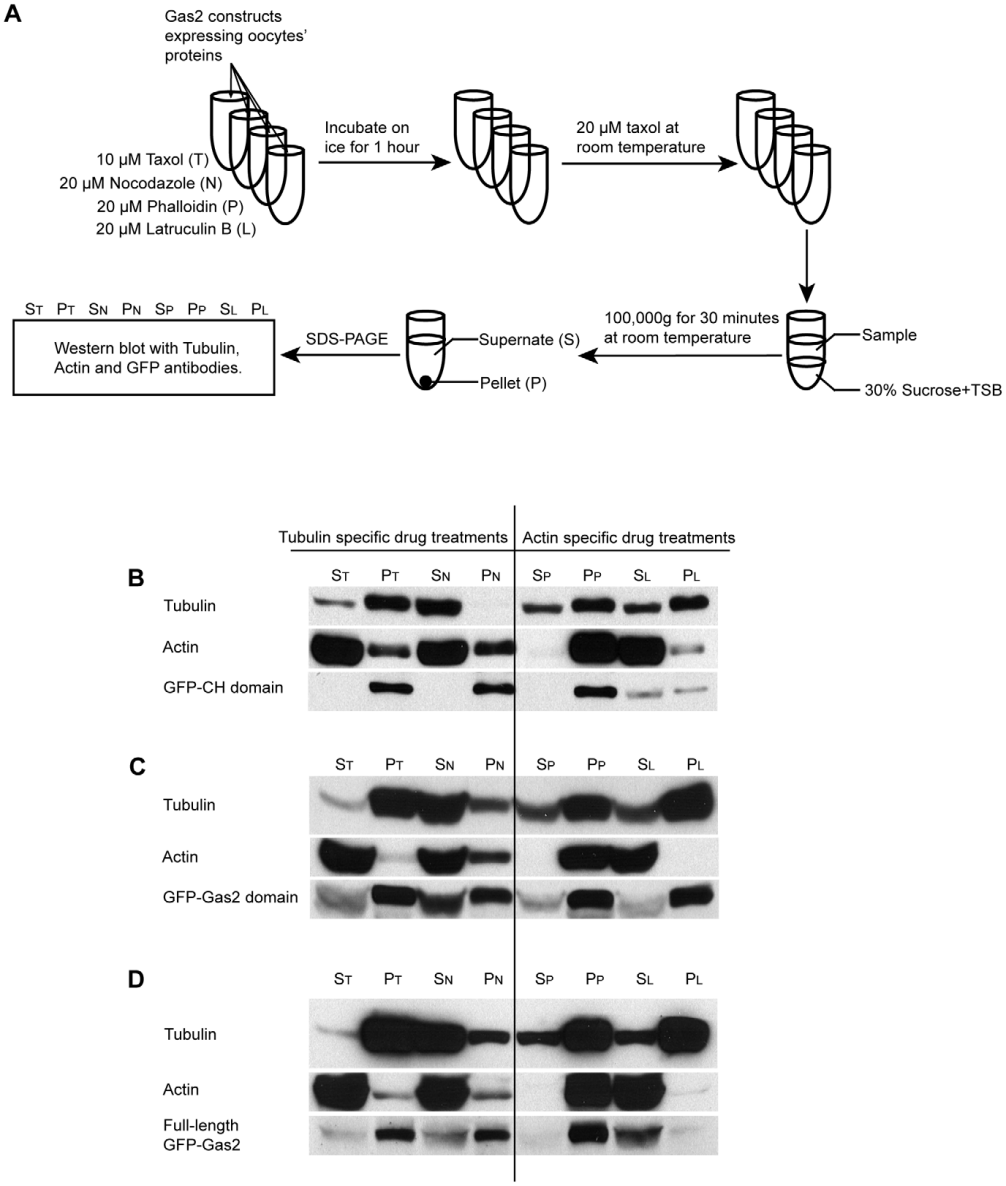
The Gas2 protein co-sediments with F-actin and microtubules. (A) The experimental flowchart of the cytoskeleton co-sedimentation assays. The samples were pre-treated with either 10 µM taxol, 20 µM nocodazole, 20 µM phalloidin, or 20 µM latrunculin B to study the cytoskeletal binding properties of Gas2. The samples were incubated on ice for one hour to de-polymerize microtubules. Taxol was added stepwise to a final concentration of 20 µM to polymerize tubulin into microtubules and samples were incubated at room temperature for one hour. Samples were loaded on the top layer of 30% sucrose solution in tubulin stabilization buffer (TSB), then centrifuged at 100,000 g for 30 minutes at room temperature. The pellets were re-suspended into the same volume as the supernate. Samples were run on SDS-PAGE for Western blot analysis. (B) The GFP-CH domain co-sediments with F-actin in the pellet. When F-actin was de-polymerized into actin monomers with latrunculin B, the CH domain was detected in the supernate (Fig. 5B: S_L_ and P_L_). (C) The GFP-Gas2 domain co-sediments with microtubule in pellets. When microtubules were de-polymerized with nocodazole, more Gas2 domain was found in the supernate compared with taxol-treated samples (Fig. 5C: S_N_ and P_N_ vs. S_T_ and P_T_). (D) The full-length GFP-Gas2 co-sediments with both F-actin and microtubules. When F-actin is de-polymerized with latrunculin B, the full-length Gas2 surprisingly remains in the supernate only. S_T_: Supernate of Taxol treatment, P_T_: Pellet of Taxol treatment; S_N_: Supernate of Nocodazole treatment, P_N_: Pellet of Nocodazole treatment; S_P_: Supernate of Phalloidin treatment, P_P_: Pellet of Phalloidin treatment; S_L_: Supernate of Latrunculin B treatment, and P_L_: Pellet of Latrunculin B treatment.

Cytoskeleton co-sedimentation assays were performed to investigate the cytoskeletal binding properties of Gas2 *in vitro*. Full-length GFP-Gas2, GFP-CH domain and GFP-Gas2 domain constructs were injected into *Xenopus* oocytes and the expressed proteins were used for co-sedimentation assays (Fig. 5A). As expected, the CH domain co-sedimented with polymerized F-actin in the pellet (Fig. 5B). When F-actin was de-polymerized into actin monomers with latrunculin B, the CH domain was detected in the supernate (Fig. 5B: S_L_ and P_L_). Similarly, the tubulin binding Gas2 domain co-sedimented with microtubules in the pellet (Fig. 5C). When microtubules were de-polymerized with nocodazole, more Gas2 domain was found in the supernate compared with taxol-treated samples (Fig. 5C: S_N_ and P_N_ vs. S_T_ and P_T_). Full-length Gas2 co-sedimented with both F-actin and microtubules (Fig. 5D). However, when F-actin was de-polymerized with latrunculin B, Gas2 remained in the supernate (Fig. 5D: S_L_ and P_L_). It was surprising to note that Gas2 did not co-sediment with microtubules in the pellet in the latrunculin B treated sample.

### The full-length Gas2 protein bundles microtubules *in vitro*

**Figure 6:**
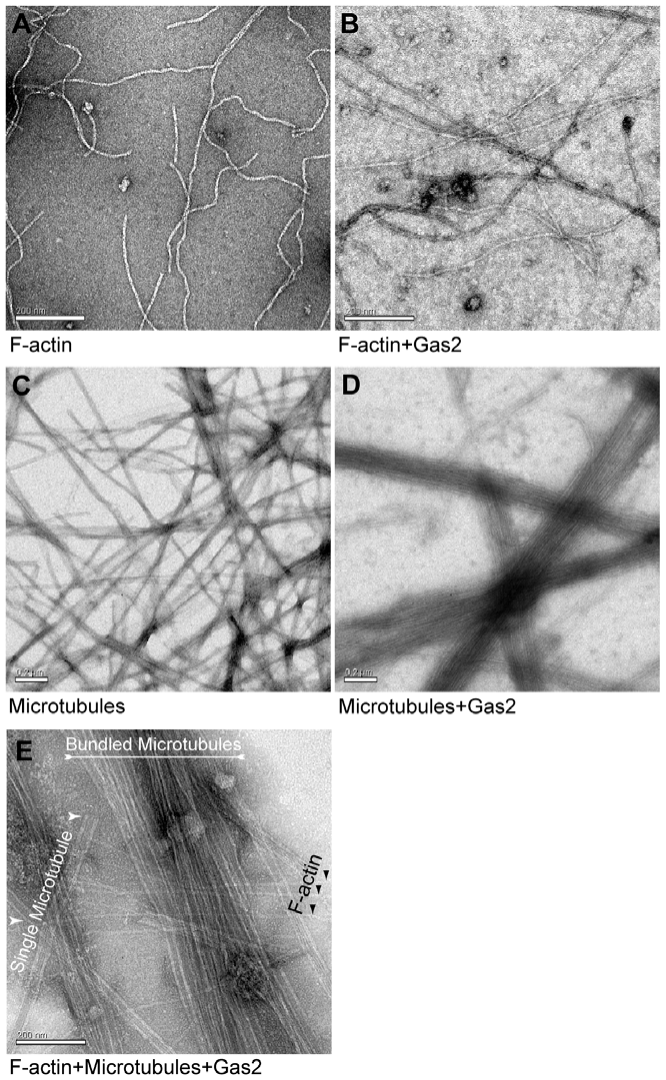
The full-length Gas2 protein bundles microtubules *in vitro*. (A) F-actin alone appears as non-organized long filaments. (B) In the presence of Gas2, F-actin remains as non-organized long filaments as in A. Bars, 200 nm. (C) Microtubules alone appear as long, randomly distributed cables. (D) Microtubules bundle together when Gas2 is present in the sample. Bars, 0.2 µm. (E) Gas2 bundles microtubules, but does not organize F-actin when microtubules, F-actin and Gas2 protein are mixed together. Bar, 200 nm. The order of Gas2 addition relative to the microtubules or F-actin has no effect on the observed results.

Gas2 interactions with F-actin and microtubules were studied at high resolution by electron microscopy (EM). The phalloidin-stabilized polymerized F-actin alone appears as non-organized long filaments (Fig. 6A). In the presence of the purified full-length Gas2 protein, F-actin remains as non-organized long filaments (Fig. 6B). Therefore, the full-length Gas2 does not possess F-actin organizing properties *in vitro*. Similarly, the taxol-stabilized polymerized microtubules appear as randomly non-organized long cables (Fig. 6C). However, in the presence of the purified full-length Gas2 protein, microtubules appear as distinct, well-organized bundles (Fig. 6D). Therefore, the full-length Gas2 protein has microtubule-organizing ability. Since the Gas2 protein has only one tubulin-binding domain, we believe Gas2 must form a protein complex in order to bundle microtubules. When F-actin, microtubules and full-length Gas2 are mixed together, Gas2 bundles microtubules, but F-actin still appears as non-organized long filaments (Fig. 6E). Similar experimental results were obtained by mixing actin or tubulin with purified full-length Gas2 protein at the beginning of their polymerization.

## Discussion

The data reported here identify Gas2 as a microtubule-bundling protein that inhibits cell division in *Xenopus* oocytes. Over-expression of the full-length Gas2 protein arrested *Xenopus* embryo cell division and resulted in multinucleated cells, indicating a failure of cytokinesis. A similar phenotype was observed upon the over-expression of the tubulin-binding Gas2 domain alone, but not when the actin-binding CH domain was over-expressed. We propose, therefore, that Gas2 inhibits cell division via its C-terminal tubulin-binding Gas2 domain.

The cytoskeleton plays a key role in cell division and cytokinesis. To investigate the cytoskeleton-binding activity of Gas2, we used a cytoskeleton co-sedimentation assay and showed that the Gas2 protein indeed co-sedimented with both F-actin and microtubules *in vitro* (Fig. 5D). Interestingly, when F-actin was de-polymerized, full-length Gas2 did not sediment with microtubule polymers in the pellet as would be expected considering the binding interaction between Gas2 and microtubules (Fig. 5D: S_L_ and P_L_). One explanation is the presence of a flexible linker region connecting the CH and Gas2 domains (Fig. 1C). This structural flexibility could permit masking of the Gas2 domain by the N-terminal region of the protein and would inhibit the microtubule-binding activity of full-length Gas2. Another possibility is that, due to the high affinity of Gas2 for F-actin, the full-length Gas2 protein binds to small, incompletely de-polymerized F-actin filaments that are too light to be pelleted and thus remain in the supernate.

Dynamic microtubules that are able to switch between polymerizing and de-polymerizing states are a necessary requirement for cell division [12]. Using electron microscopy we have shown that Gas2 bundles purified microtubules into higher-order structures *in vitro,* indicating a direct Gas2-microtubule interaction (Fig. 6D). The full-length Gas2 microtubule-bundling ability is preserved when F-actin is present in the same sample, but no F-actin-microtubule cross-linking has been observed *in vitro* (Fig. 6E). We hypothesize that Gas2-microtubule bundling ability inhibits microtubule dynamics and thereby causes cell division arrest and cytokinesis failure in *Xenopus* embryos.

To investigate the mechanism of Gas2-induced cell division arrest and cytokinesis failure, we used a wound-induced contractile array assay in *Xenopus* oocytes, which is also a model of cytokinesis [10]. This assay permitted us to study Gas2-cytoskeletal interactions during microtubule-dependent actomyosin array formation and contraction *in vivo* [10]. Oocyte wound healing and cytokinesis are highly dynamic processes that require coordination between the actin and microtubule cytoskeletons [13]. We have shown that Gas2 possesses both actin- and tubulin-binding properties *in vitro*; therefore, we propose that Gas2 functions as a structural cross-linking protein between these two cytoskeleton systems. When oocytes are treated with taxol to stabilize microtubules prior to wounding, an abnormal double ring of actin forms around the wound border [2]. Staining with phalloidin for F-actin showed that the internal ring is composed of contractile F-actin, while staining for actin monomers showed that the outer ring is formed by *de novo* actin polymerization [2], [13]. Interestingly, we found that when full-length Gas2 or its Gas2 domain was over-expressed, oocytes developed the abnormal double actin ring phenotype upon wounding. Thus, Gas2 mimics the taxol-treatment phenotype, suggesting that Gas2 has microtubule-stabilizing activity. The Gas2-induced double rings can be rescued by de-polymerizing microtubules with nocodazole (Fig. 4L–O) perhaps because the Gas2 protein binds dynamically to microtubules. Full-length Gas2 co-localizes with the actin rings, presumably by binding actin via its CH domain (Fig. 4H–K). It is interesting to note that the Gas2 domain alone also forms a ring structure and localizes between two actin rings during the wound healing (Fig. 4T–W), but it does not overlap with either actin ring since it has no actin-binding domain.

In summary, we have demonstrated that Gas2 can inhibit cell division in *Xenopus* embryos. We have also provided evidences that Gas2 has the ability to change microtubule dynamics and bundle microtubules *in vitro*. We believe that the microtubule-bundling ability of Gas2 causes *Xenopus* embryo cell division arrest and the formation of the abnormal double actin rings in oocyte wound healing.

*Drosophila* contains a spectraplakin family protein, Short stop (Shot), that modulates microtubule dynamics via its Gas2 domains [14]. Röper and Brown showed that Shot has a Gas2 domain at its C-terminus and is required for organizing microtubules in a branched membrane structure called the fusome in meiotic cysts. They demonstrated that, in the absence of Shot, microtubules did not assemble, and oocytes failed to become specified. The authors postulated that Shot elicits this effect via its Gas2 domain influencing microtubule dynamics. Similarly, Pines et al. showed that a Gas2-like protein, Pickled eggs (Pigs) co-localizes with both F-actin and microtubules in the giant epithelial cells in *Drosophila* [15]. Pigs is postulated to facilitate cytoskeleton rearrangements by binding and stabilizing F-actin and microtubules. Consistent with our data, it is likely that Gas2 has conserved cytoskeleton-binding properties in different species. Although a recent paper by Stroud et al., showed that the GAR domain (the other name of Gas2 domain) of Gas2-like 3 protein is not essential for its localization to microtubules and does not directly interact with microtubules *in vitro*, this may be due to the large C-terminus domain which is present in Gas2-like 3 protein, but not in the Gas2 protein [16]. This domain may directly impact on protein functions including the ability of Gas2-like 3 to bind microtubules.

Several studies have provided evidence suggesting that Gas2 activity is mis-regulated in cancer. Human *gas2* is located on the short arm of Chromosome 11 and genetic rearrangements or deletions of this region are frequently found in tumors and sporadic human cancers [17], [18], [19], [20]. The analysis of oncogene transformed cells *v-fos*, *v-myc*, *v-ras* and *v-src* showed that the expression of Gas2 fails to increase in response to serum starvation [6]. Furthermore, proteomic studies have identified Gas2 in normal rat liver cells, but not in the rat liver tumors [21]. Finally, translation initiation factor eIF4E binding proteins have been shown to control p53-dependent senescence by regulating Gas2 translation [22]. In light of this, a better understanding of the role of Gas2 functions in cell division and how Gas2 is regulated will aid the development of improved cancer therapies.

## Materials and Methods

### Experimental animals

*Xenopus laevis* frogs were bought from Nasco (Fort Atkinson, WI, USA). The experiments were conducted by trained skilled personnel and were approved by McGill University Animal Care Committee (Protocol #: 4858 to C.A.M.).

### Bioinformatics analysis

*Homo sapiens* (human) [O43903], *Canis familiaris* (Dog) [F1PBV2], *Mus musculus* (house mouse) [P11862], *Bos taurus* (Bovine) [A8E4Q5], *Gallus gallus* (Chicken) [F1NSM4] and *Xenopus tropicalis* (Silurana tropicalis) [ENSXETP00000005555] Growth-arrest-specific protein 2 (Gas2) amino acid sequences were obtained from NCBI (http://www.ncbi.nlm.nih.gov), Uniprot (http://www.uniprot.org/) and Ensembl (http://www.ensembl.org/Xenopus_tropicalis) websites. They were compared the similarity with ClustalW (http://www.ebi.ac.uk/Tools/clustalw2/index.html). Phylogenetic tree was also derived by aligning the different species' amino acids with ClustalW. Gas2 domain information was acquired from http://pfam.sanger.ac.uk/protein?acc=P11862.

### Cloning

DNA constructs were generated by PCR and cloned into pCS2-eGFP vector (a kind gift from Prof. Bill Bement, University of Wisconsin-Madison, USA). The full-length Gas2 (cDNA clone MGC:18565) was cloned with N-terminus GFP tag by using the forward primer 5′-ATGTGCACTGCCCTGA-3′ and reverse primer 5′-TCATTTAATCTCCTTCTTAGCCTTG-3′. Gas2 CH domain (with N-terminus GFP tag) was cloned with the forward primer 5′-TGCACTGCCCTGAGCC-3′ and reverse primer 5′-TCATCCAGTACTCTTCTTTCCTGAAG-3′; and its Gas2 domain (with N-terminus GFP tag) was cloned with the forward primer 5′-AGGTATGGTGTGGAGCCTCCT-3′ and reverse primer 5′-TCATTTAATCTCCTTCTTAGCCTTGT-3′. All clones were sequenced to ensure the sequence correctness.

### Western blot

GFP tagged Gas2 constructs were injected into *Xenopus* oocytes nuclei and oocytes were incubated for 48 hours to allow for relative protein expression. The oocytes were then sonicated in phosphate buffered saline (PBS: 137 mM NaCl, 2.7 mM KCl, 10 mM Na_2_HPO_4_ and 2 mM KH_2_PO_4_ at pH 7.4) with ultrasound sonicator, and samples were centrifuged at 30,000 g for 10 minutes at 4°C. Supernatants were collected for Western blot analysis. Protein assays were performed using the BCA protein assay kit (Thermo). Protein samples were run on a 12% SDS-PAGE gel and transferred to 0.45 µm Trans-Blot nitrocellulose paper (Bio-Rad), which was blocked with blotto buffer (5% non-fat milk in PBS) after transferring. After washing in 0.1% Tween-20 (Fisher) in PBS (PBS-T), monoclonal mouse GFP antibodies (Roche, Clone 7.1 and 13.1) were used at 1∶3000 dilution in blotto buffer incubated at room temperature for 2 hours. The non-specific antibody binding was washed 3 times with PBS-T. Primary antibodies were detected using goat anti-mouse IgG antibodies conjugated to horse radish peroxidase (HRP) (Molecular Probes) at 1:3000 dilution in PBS at room temperature for 1 hour, and followed by 3 times washing with PBS-T. Detection was performed using the ECL method.

### Protein expression and purification

Gas2 (cDNA clone MGC:18565) was cloned into pTrcHis2B vector with 6X His tag at N-terminus using the forward primer 5′-ATGTGCACTGCCCTGA-3′ and reverse primer 5′-TTTAATCTCCTTCTTAGCCTTG-3′. Basically, transform pTrcHis2B-Gas2 construct into DH5α *Escherichia coli* to express full-length Gas2 protein. Protein was collected with Probond nickel-chelating resin (Invitrogen). The elution protein solution was kept in a dialysis bag (Spectra/Pov molecularporous membrane tubing, MWCO 12–14 kDa, Fisher 08-667B) in PBS at 4°C overnight to remove imidazole. The protein sample was re-concentrated with Microcon centrifugal filter devices (Millipore: YM-10), and then was aliquoted into small volume and frozen in liquid nitrogen. Samples were stored at −80°C for further use. The protein purity was tested by Coomassie-stained SDS-PAGE, and no significant additional bands were observed.

### *Xenopus laevis* eggs collection and fertilization

Human chorionic gonadotropin (hCG) was injected into 3 adult *Xenopus* females (1000 U/ml, 0.5 ml per frog) to induce ovulation. Eggs were harvested in glass Petri dish by gently squeezing the female. Eggs were fertilized by adding up a piece of a testis in the Petri dish and swirling gently. Ninety minutes later, 40 ml of 2% cysteine (Sigma) at pH 8.0 was added for 5 minutes to de-jelly embryos. Cysteine was poured of and replaced with Marc's modified ringers (MMR: 100 mM NaCl, 2 mM KCl, 1 mM MgCl_2_, 2 mM CaCl_2_ and 5 mM HEPES at pH 7.5). Embryos were incubated at 16°C. They develop more rapidly at room temperature.

### Microinjection

Microinjection needles (10 µl Drummond microdispenser, Drummond Scientific Company) were pulled by Flaming/Brown micropipette puller (Model P-97, Sutter Instrument Company). Five nl of 12 µg/µl bovine serum albumin (BSA) in PBS solution or the same amount of purified full-length Gas2 protein in PBS solution was injected into one cell of 2-cell stage *Xenopus* embryos. Injections were performed using a Model PLI-100 Pico-Injector (Harvard Apparatus).

### Live imaging movies

The movies were recorded at a rate of 1 frame per minute with Zeiss Axioskop 2 microscope. Zeiss AxioCam MRm color camera and Plan-Neofluar 2.5X/0.075 N.A. objective lens were used. The images were modified with Volocity version 5.3.0 software (PerkinElmer) and played at 10 frames per second in Apple QuickTime (*.mov) format.

### Microinjection experiments results statistics tabulation

The post-injected embryos were examined for the arrested cell division phenotype under a stereo microscope. Statistics results were charted with Microsoft Office Excel software, and analysis was performed using Student's t-test.

### Cyro sample preparation and immunofluorescence

One hundred pg each of GFP, full-length GFP-Gas2, GFP-CH domain or GFP-Gas2 domain constructs were injected into one cell of 2-cell stage embryos cytoplasm. The injected embryos were incubated in MMR solution and developed until Stage 10 or 11 before cryo preparation. Embryos were fixed and embedded in 15%, then 25% fish gelatin (Norland). They were frozen on dry ice before cutting. Ten µm thickness of cryo sections were cut in −20°C cyrotome (Leica), and collected on Superfrost plus slides (Fisher). A polyclonal rabbit Gas2 antibody (against peptide 78–99 KLH conjugation, provided by Prof. Jacque Paiement at University of Montreal) at 1∶300 dilution, or a polyclonal rabbit GFP antibody (Molecular Probes) at 1∶200 dilution, and a monoclonal alpha tubulin antibody (Sigma, DM1A) at 1∶200 dilution were used. A goat-anti rabbit antibodies coupled to Alexa Fluor 488 (Molecular Probes) at 1∶200 dilution, and a goat-anti mouse antibodies coupled to Alexa Fluor 546 (Molecular Probes) at 1∶200 dilution were used for the staining. Nuclei were stained with DAPI (Molecular Probes) (0.5 g/ml) in PBS for 10 minutes. The yolk auto-fluorescence was quenched with 0.2% Eriochrome black (Sigma) in PBS for 10 minutes. Finally, the slides were mounted with ProLong Gold anti-fading reagent (Molecular Probes) and covered with cover slips (Fisher: 12–545M) for microscopy examination.

### *Xenopus laevis* ooctyes collection and preparation

Oocytes were surgically removed from an anesthetized adult *Xenopus laevis* female, as previously described [11] and stored at 16°C in oocyte ringer-2 solution (OR-2: 82.5 mM NaCl, 2.5 mM KCl, 1 mM CaCl_2_, 1 mM MgCl_2_, 1 mM Na_2_HPO_4_, 5 mM HEPES at pH 7.4). Oocytes were treated with 0.2% collagenase (Type 2) OR-2 solution with gentle rotation at room temperature for 1 hour. Oocytes were washed after the treatment until the brown color collagenase was removed. The washed oocytes were kept at 16°C in OR-2 solution for at least 3 hours for recovery. Healthy oocytes have uniform animal and vegetal hemispheres. StageVI oocytes were manually chosen and de-folliculated with Watchmaker's forceps (Dumont #5) in a Petri dish containing OR-2 solution. This step is critical for cortex wound healing experiments.

### Pharmacological perturbations and oocyte wounding

Oocytes were incubated in fresh 10 µM taxol (Sigma) or 20 µM nocodazole (Calbiochem) OR-2 solution for 1 hour at 16°C. The wounding experiments were performed in the same solutions. Oocytes were placed in a Petri dish fitted with a nylon grid to position the animal pole facing upwards. They were then wounded in the animal pole with a microinjection needle (cut to outside with a diameter of approximate 150 µm). Cells were incubated for about 5 minutes in OR-2 solution for recovering before being placed in fix solution.

### *Xenopus laevis* embryos and oocytes immunofluorescence

The protocol was the same as previously described [11]. Briefly, embryos or wounded oocytes were fixed and incubated with Alexa Fluor 568 phalloidin (6.6 µM, at 1∶200 dilution) (Molecular probes) and monoclonal alpha tubulin antibody (1∶200 dilution) (Sigma, DM1A). Samples were then re-probed with Alexa Fluor 568 phalloidin for F-actin and Alexa Fluor 647 goat anti-mouse IgG antibody (1∶200 dilution) (Molecular probes) for alpha tubulin. Finally, samples were carefully placed in the center of a high vacuum grease (Dow Corning Co.) mounted ring on a microscope slide plain (Fisher), and covered with a microscope cover glass #1.5 thickness (Fisher) for microscopy examination.

### Confocal fluorescence microscopy

All confocal images were collected using Zeiss 510 LSM Meta confocal microscope at Cell Imaging and Analysis Network at McGill University. The samples were examined with Plan-Neofluar 25X/0.8 N.A. Immersion Correction DIC or Plan-Apochromat 63X/1.4 N.A. oil DIC objective lens. DAPI (Molecular probes) staining was visualized with 405 nm blue diode laser and band pass (BP) 420–480 filter; GFP signal was visualized with 458 nm Ar ion laser and BP 505–530 filter; Alexa Fluor 568 was visualized with 543 nm HeNe green laser and BP 560–615 filter; and Alexa Fluor 647 was visualized with 633 nm HeNe red laser and long pass (LP) 650 filter. Z-sections of varying depths were stacked into projection images with maximum intensity to allow visualization of details.

### Cytoskeleton co-sedimentation assays

The full-length GFP-Gas2, GFP-CH domain and GFP-Gas2 domain constructs were injected into *Xenopus* oocytes and oocytes were incubated at 16°C for 48 hours to allow for relative proteins expression. The expressing proteins were collected by sonication and following by centrifugation. Only the cytosolic layers were used for co-sedimentation assays. The samples were separately pre-treated with 10 µM taxol (Sigma), 20 µM nocodazole (Calbiochem); and 20 µM phalloidin (Calbiochem), and 20 µM latrunculin B (Calbiochem) to investigate Gas2 cytoskeleton binding properties. The mixture samples were incubated on ice for 1 hour to de-polymerize microtubules, and then taxol was step wisely added to the final concentration 20 µM to polymerize tubulin into microtubules filaments at room temperature for one hour. Samples were loaded on the top layer of 30% sucrose in tubulin stabilization buffer (TSB: 1 mM EGTA, 5 mM MgCl_2_, 80 mM K-PIPES at pH 7.0), and then they were centrifuged at 100,000 g for 30 minutes at room temperature. The pellets were diluted into equal volume as the supernatant samples. Samples were run on 10% SDS-PAGE for Western blot analysis. The following antibodies were used for the Western blot analysis: the monoclonal mouse tubulin antibodies (Sigma, DM1A) were used at 1∶3000 dilution; the polyclonal rabbit actin antibodies (Biomedical Technologies Inc. BT-560) were used at 1∶3000 dilution; the monoclonal mouse GFP antibodies (Roche, Clone 7.1 and 13.1) were used at 1∶3000 dilution. Primary antibodies were detected using IgG antibodies conjugated to HRP (Molecular Probes) at 1∶3000 dilution by ECL method.

### Electron microscopy

Purified actin (AKL99-A) and tubulin (TL238-A) were bought from Cytoskeleton Inc. (Denver, CO.). Actin was first dissolved in the general actin buffer (5 mM Tris-HCl at pH 8.0, 0.2 mM CaCl_2_, 0.2 mM ATP), and then phalloidin (Calbiochem) was added up to final concentration 20 µM to polymerize actin into F-actin. Tubulin was first dissolved into general tubulin buffer (80 mM PIPES at pH 6.9, 2 mM MgCl_2_, 0.5 mM EGTA, 1 mM GTP), and then 20 µM final concentration taxol (Sigma) was used to polymerize tubulin into microtubules. The purified full-length Gas2 protein in PBS was mixed with F-actin or microtubules or both for EM examination. A similar experiment was done by adding the full-length Gas2 to actin or tubulin samples before their polymerization.

For each EM sample, 5 µl protein sample was placed on a glow discharged pre-carbon coated copper grid for 1 minute, and then was replaced by 5 µl 1% uranyl acetate for 1 minute treatment. Uranyl acetate was carefully removed and the treated grid was left by air-dried. Samples were examined in an FEI Tecnai 12 electron microscope, and digital images were taken by a Gatan 792 Bioscan wide angle multiscan CCD camera.

## Supporting Information

Video S1 **BSA-injected** ***Xenopus*** **embryos cell division time-lapse movie.** The movie was recorded at 1 frame per minute and plays at 10 frames per second.
(MOV)

Video S2 **Gas2-injected** ***Xenopus*** **embryos cell division time-lapse movie.** The movie was recorded at 1 frame per minute and plays at 10 frames per second.
(MOV)
